# Psychological therapy for mood instability within bipolar spectrum disorder: a randomised, controlled feasibility trial of a dialectical behaviour therapy-informed approach (the ThrIVe-B programme)

**DOI:** 10.1186/s40345-021-00226-4

**Published:** 2021-07-01

**Authors:** Kim Wright, Alyson L. Dodd, Fiona C. Warren, Antonieta Medina-Lara, Barnaby Dunn, Julie Harvey, Mahmood Javaid, Steven H. Jones, Christabel Owens, Rod S. Taylor, Deborah Duncan, Alexandra Newbold, Shelley Norman, Faith Warner, Thomas R. Lynch

**Affiliations:** Washington Singer Labs., University of Exeter Department of Psychology, Perry Road, Exeter, EX4 4QG UK

**Keywords:** Bipolar disorder, Dialectical behavior therapy

## Abstract

**Background:**

A subgroup of those with bipolar spectrum disorders experience ongoing mood fluctuations outside of full episodes. We conducted a randomised, controlled feasibility study of a Dialectical Behavioural Therapy-informed approach for bipolar mood fluctuations (Therapy for Inter-episode mood Variability in Bipolar [ThrIVe-B]). Our study aimed to examine the feasibility and acceptability of a future definitive trial evaluating the clinical and cost effectiveness of the ThrIVe-B programme. Participants were required to meet diagnostic criteria for a bipolar spectrum disorder and report frequent mood swings outside of acute episodes. They were randomised to treatment as usual (control arm) or the ThrIVe-B intervention plus treatment as usual (intervention arm). Follow-up points were at 3, 6, 9 and 15 months after baseline, with 9 months as the primary end point. To evaluate feasibility and acceptability we examined recruitment and retention rates, completion rates for study measures, adverse events and feedback from participants on their experience of study participation and therapy.

**Results:**

Of the target 48 participants, 43 were recruited (22 in the intervention arm; 21 in the control arm), with a recruitment rate of 3.9 participants per month. At 9 months 74% of participants engaged in research follow-up assessment, exceeding the pre-specified criterion of 60%. There were no serious concerns about the safety of the research procedures or the intervention. On one of the four candidate primary outcome measures, the 95% CI for the between-group mean difference score excluded the null effect and included the minimal clinically important difference, favouring the intervention arm, whilst on no measure was there evidence of deterioration in the intervention arm relative to the control arm. Attendance of the intervention (50% attending at least half of the mandatory sessions) was below the pre-specified continuation criterion of 60%, and qualitative feedback from participants indicated areas that may have hampered or facilitated engagement.

**Conclusions:**

It is broadly feasible to conduct a trial of this design within the population of people with frequent bipolar mood swings. Changes should be made to the therapy to increase uptake, such as simplifying content and considering individual rather than group delivery.

*Trial registration* ISRCTN: ISRCTN54234300. Registered 14th July 2017, http://www.isrctn.com/ISRCTN54234300

**Supplementary Information:**

The online version contains supplementary material available at 10.1186/s40345-021-00226-4.

## Background

Within bipolar spectrum disorders, ongoing instability of mood (henceforth referred to as bipolar mood instability [BPMI]) is characteristic of Cyclothymic Disorder, yet can also be experienced by individuals with Bipolar disorder I or II outside of acute episodes of mania or depression (Bonsall et al. 2012). BPMI may be an important target for intervention in its own right: as well as Cyclothymic Disorder being a risk factor for future episodes of mania and depression (Akiskal et al. 1977), BPMI between episodes is associated with increased psychiatric comorbidity, reduced psychosocial functioning and lower self-reported quality of life (Faurholt-Jepsen et al. 2019; Henry et al. 2008; Gershon and Eidelman 2015; Strejilevich et al. 2013).

Trials of medication for Bipolar disorders rarely target BPMI specifically and consequently there is no generally accepted pharmacological treatment strategy for this aspect of the condition. Similarly, trials of psychological therapies for Bipolar disorders have tended to focus upon treating acute depression or reducing risk of relapse rather than reducing mood instability. Whilst a small number of studies have tested psychological interventions for Cyclothymic Disorder or given consideration to BPMI within Bipolar disorder (Fava et al. 2011; Hales et al. 2018; Totterdell and Kellett 2008; Totterdell et al. 2012), none of these have tested an intervention specifically developed for BPMI across the Bipolar Spectrum. Here we report a randomised controlled feasibility study of such an approach.

Our approach is informed by Dialectical Behaviour Therapy (Linehan 1993) which was originally developed for another patient group with ongoing instability of mood, those with a diagnosis of Emotional Unstable Personality Disorder (EUPD). DBT teaches skills both in acceptance of situations and emotional responses, and in selection of adaptive behavioural responses to emotional and situational triggers, and has been found to be effective in EUPD (Cristea et al. 2017). Whilst versions of DBT have been explored within those with Bipolar disorder in an open trial and two pilot randomised controlled trials (Goldstein et al. 2007, 2015; Dijk et al. 2013) none of these have specifically targeted those experiencing BPMI.

To address this gap we developed the ThrIVe-B programme (Therapy for Inter-episode mood Variability in Bipolar) as a bespoke approach for those with BPMI. In this approach participants are invited to reflect upon their behavioural response to extreme mood and activation states day-to-day, modifying this where necessary, congruent with evidence that unhelpful behavioural response to mood is associated with bipolar symptom exacerbation (Lam et al. 2001; Palmier-Claus et al. 2016). In DBT this is achieved by building mindful awareness skills and through giving participants a framework through which to appraise their emotional responses and to develop alternative ways of relating to and managing these. In ThrIVe-B we further developed this process with respect to hypomanic and depressive feelings. ThrIVe-B also attended to triggers of mood dysregulation: circadian rhythm disruption has been found to precede the onset of Bipolar episodes (Takaesu 2018) whilst social support appears to be associated with a more favourable course of symptoms (Cohen et al. 2004; Johnson et al. 1999) therefore we utilised elements of DBT that promote stable routine and interpersonal problem-solving and communication, and developed elements around mobilising social support.

Initial evaluation of the ThrIVe-B programme in an uncontrolled feasibility study (N = 12) (Wright et al. 2020) found it to be acceptable to recipients: 75% completed therapy. Consistent with potential for clinical benefit, reliable improvement in depression, anxiety and general mental health symptoms was observed in least half the participants, with very few instances of reliable deterioration on any measure. Participant feedback supported this, and congruent with the key putative mechanism of change, participants described gaining the ability to step back from intense feelings and make better choices. There were no trial-related serious adverse events or concerns about therapy safety.

To resolve uncertainties in the design and delivery of a future efficacy trial, the present study was a randomised, controlled feasibility study across two sites (Devon and Cumbria). Our objectives were to: (i) establish recruitment pathways and trial teams in two sites; (ii) establish the number of participants initially identified, approached, consented, randomised and completed; (iii) establish the acceptability and experience of the trial process to participants, including randomisation and completion of outcome measures; (iv) further assess the acceptability of the treatment via qualitative interviews and, based on input from trial participants and clinicians, to further refine and develop the treatment manual and the procedures for training, supervising and assessing the competence of trial therapists; (v) assess the performance of selected candidate primary outcome measures for a future definitive trial with respect to level of acceptability to participants and participant-perceived relevance and value; (vi) measure data completeness at follow-up, SD of the likely primary outcome measure, and describe the variability of the comparator condition, treatment as usual (TAU), across individuals and sites; (vii) pilot a measure of resource use and to assess the feasibility and acceptability of candidate health outcome measures for economic evaluations; and (viii) identify, measure and cost the resources used and needed to deliver the intervention.

We also aimed to evaluate whether the following continuation criteria were met prior to planning a future definitive trial: (i) trial participation does not lead to serious negative consequences (unexpected serious adverse reaction) for participants; (ii) any serious concerns about the acceptability and feasibility of the trial procedures can be rectified prior to a full trial; (iii) follow-up data at 9 months are available for at least 60% of participants; (iv) at least 60% of patients in the intervention group complete treatment (attend at least 50% of possible sessions).

## Method

### Trial design

We conducted a feasibility study with a two-arm, randomised, parallel, controlled trial design. Participants were randomised in a 1:1 ratio to TAU only (control arm) or TAU plus the ThrIVe-B programme (intervention arm). Outcome measures were administered at baseline and at 3, 6, 9 and 15 months after randomisation. The primary end point was predetermined to be at 9 months. The full trial protocol is reported elsewhere (Wright et al. 2018).

A panel of individuals with lived experience of mood disorders advised on study design and delivery throughout the trial.

### Participants

Participants were required to be aged 18 years or older, with a diagnosis of BD (I, II, other specified BD) or Cyclothymic Disorder, according to the criteria in the Diagnostic and Statistical Manual of Mental Disorders, Fifth Edition (DSM-V) (American Psychiatric Association 2013), and to have a period of at least 2 days during which symptom criteria for hypomania/mania were met. They were required to have current BPMI, defined as either meeting DSM-V criteria 1 and 2 for Cyclothymic Disorder, or having a score of at least 1.3 [the mean for individuals with Bipolar disorder in previous research (Henry et al. 2008)] on the bipolar subscale of the short form of the Affective Lability Scale (ALS) (Oliver and Simons 2004). Participants had to be willing to engage in psychological therapy that focusses primarily on ongoing mood instability and its consequences, and be willing and able to attend scheduled group therapy sessions. Sufficient competency in English to be able to complete study measures without the need for translation was required, as was registration with a primary care physician in the study catchment area. Exclusion criteria included current substance dependence, other ongoing psychological therapy for BD at study entry, lack of capacity to consent to treatment or research participation, and acute mania or depression (DSM-V). Participants engaging in frequent, significant self-harming behaviour, at high risk of suicide, or posing a significant risk to other group members were not eligible. Initially patients receiving ongoing, co-ordinated care in secondary mental health services were excluded from participation because the ThriVe-B programme was aimed at those at the interface between primary and secondary care, however this criterion was removed at month 9 of the 11 month recruitment phase to facilitate recruitment.

Participants were recruited from two sites in the United Kingdom, Cumbria and Devon. Both are relatively rural however Cumbria has a population density less than half that of Devon’s. Recruitment was via primary care services, secondary care mental health services and self-referral. Following an expression of interest, participants providing written agreement to be contacted by the study team were sent information about the study and invited to attend a baseline eligibility assessment interview. Those not referred from secondary care (and thus who had not recently participated in a specialist Psychiatric assessment) completed a telephone screening interview prior to the eligibility assessment to increase the likelihood that those invited for a time-consuming face-to-face assessment would be eligible. At the eligibility assessment written, informed consent was sought, eligibility criteria were checked and baseline measures completed.

### Planned sample size

On the basis of our previous work (Wright et al. 2020) we estimated an attrition rate of 17% with respect to the 9 month primary end point. A sample of 48 would allow estimation of this level of attrition for a future definitive trial with a precision of ± 15% with 95% certainty. The 48 participants (24 per arm) would represent recruitment to three ThrIVe-B groups at full capacity (8 per group: 2 in Devon and 1 in Cumbria).

### Intervention

The intervention (ThrIVe-B) was delivered in addition to TAU. The content of the ThrIVe-B programme is described in detail in the logic model in Additional file 1. In brief, modules covered Mindful Awareness, Day to Day Mood Regulation. Tolerating Intense Moods, and Wise Relationships The programme consisted of 15 weekly group meetings and up to eight individual sessions of up to 45 min held either face-to-face or by telephone, according to participant preference for modality, number and duration. All participants were expected to attend the initial and final individual meeting. There was a group “booster” session 3 months after the final group meeting, and midway through the programme participants were offered a “supporters” session which friends or family could attend. To help generalisation of learning, participants were given a folder of written materials. In addition we used a custom-built smartphone application (‘ThrIVe-B app’) to prompt participants to use skills at times of extreme mood, between therapy sessions. The app asked participants to rate their mood from − 10 to + 10 each day between 0 and 10 times, with number of ratings per day determined by the user. Users specified ‘high’ and ‘low’ mood thresholds and when these were surpassed, users saw a feedback screen containing advisory messages they had pre-programmed either alone or with therapist support, which could contain techniques from the therapy programme (see Additional file 2). A version of this app, with reduced functionality, was also used to monitor mood stability over 7-day periods at baseline and at follow-up for all participants.

Therapy was delivered by four qualified mental health professionals with previous specialist training in cognitive behavioural therapy and related approaches (two therapists per group). A 5 day bespoke, face-to-face training programme was provided by the main author of the ThrIVe-B therapy protocol, which covered background information about Bipolar disorder, the therapeutic stance within DBT-informed approaches, therapy components, and skills in group facilitation. Weekly supervision occurred throughout therapy delivery. Before each group session participants completed the Beck Depression Inventory (Beck et al. 1961) and Altman Scale for Rating Mania (Altman et al. 1997).

Participants in the TAU arm received their usual NHS care, with no stipulation from the trial regarding the minimum constituents of this. In addition, in order that the two arms were broadly equivalent in time spent using the app between assessment points, those in the TAU arm were invited to use it for mood monitoring only (no additional therapeutic functionality) between months 3 and 9. As per the intervention arm, participants could set number of alerts per day from 0 to 10. Other than the experimental intervention no treatments were withheld.

### Outcomes

Primary outcome measures. Numbers of patients identified, approached, consented, randomised and completed were used to assess feasibility, as was participant attrition from the trial and from treatment. Acceptability was assessed by questionnaires at baseline, 9 month follow-up and on ineligibility or withdrawal from the study. Items with quantitative ratings at 9 month follow-up were rated on a five point scale and asked about acceptability and satisfaction (anchored from “not at all” to “extremely”) and likelihood of recommending the trial to eligible friends and family. To assess patient valuation of outcomes, at the 9-month follow-up point participants were asked to rank the four candidate outcome measures (see below) and a measure of anxiety symptoms, the Generalised Anxiety Disorder 7-item scale (GAD-7) (Spitzer et al. 2006) in order of priority, in terms of where they would most want to see change following psychological treatment. Participants were also asked to estimate the smallest change on each measure that would feel meaningful.

A subset of 14 participants from both sites took part in a semi-structured interview exploring their experiences of the research study and therapy in more detail. Referring clinicians were asked for their views of the therapy and research process using a brief survey form, and ThrIVe-B therapists took part in an interview about their experiences and views of the therapy, training and supervision.

Adherence to treatment was measured in terms of number of therapy sessions attended; because some individual sessions were optional, “possible sessions” (continuation rule 4) was defined as all 15 group sessions, booster session and initial and final individual sessions (18 sessions in total).

The ThrIVe-B app was used to gather data on momentary mood in order to assess mood stability. All participants were invited to use the app for 1 week at baseline and at 9-month follow-up, to give comparable 1 week periods of mood stability data that did not overlap with periods of therapeutic app use. Mood stability measured by the ThrIVe-B app is a potential secondary outcome in a definitive trial, therefore we tested the feasibility and acceptability of gathering these data, as indicated by completion rates and participant feedback.

Secondary outcome measures. The following were candidate primary outcome measures for a future definitive trial and were completed at baseline and all follow-up points: the Patient Health Questionnaire 9-item scale (PHQ-9) (Spitzer et al. 1999) which measures depressive symptoms; the short-form Affective Lability Scale; the Bipolar Recovery Questionnaire (BRQ) (Jones et al. 2013); and the brief Quality of Life in Bipolar disorder scale (QoL-BD) (Michalak et al. 2010). Participants also completed the GAD-7. At baseline, 9 and 15 months, the Bech Mania Rating Scale (BMRS) (Bech et al. 1978), the Hamilton Depression Rating Scale (HDRS) (Hamilton 1986) (both observer-rated), and the Brief Adherence Rating Scale for medication adherence (BARS) (Byerly et al. 2008) were completed, as was relevant sections of the Structured Clinical Interview for DSM-V (First et al. 2014). This was used both to assess eligibility criteria, and to identify affective episodes during the follow-up period.

To assess feasibility of health outcome measures for a future cost-effectiveness analysis was assessed with two measures: the EuroQoL 5-dimension 3-level (EQ-5D-3L) (Oppe and Devlin 2007),^1^ and the 36-item Short Form Health Survey (SF-36) (Garratt et al. 1993), these were administered at baseline and follow-up points. In addition, participants were asked to complete a resource use questionnaire (last 6 months or since last assessment point) for assessing the feasibility of collecting accurate health and social service use. Finally information on resource use and costs of delivering the ThrIVE-B programme were also collected.

#### Quantitative process measurement

Measurement of putative mechanisms of change was included in order to inform measurement in a definitive trial. These were completed at baseline and all follow-up points. We included measures of impulsive behavioural response to high and low mood (Positive and Negative Urgency, Premeditation, Perseverance, Sensation-Seeking impulsive behaviour scales [UPPS-P] (Cyders et al. 2007); Behavioral Activation in Depression Scale [BADS] (Kanter et al. 2007)) as this is a key process that ThrIVe-B aims to disrupt. In DBT this is achieved in part by building mindful awareness skills, hence we measured this using the Kentucky Inventory of Mindfulness Skills (KIMS) (Baer et al. 2004). We assessed social rhythm stability using the adapted Social Rhythm Metric (SRM) (Monk et al. 1990) and emotional problem solving using the Means-Ends Problem Solving task (MEPS: baseline, 9 and 15 months only) (Kehrer and Linehan 1996).

The schedule for administration of study measures is shown in Additional file 3.

### Procedure

Prior to conducting the trial ethical approval was obtained from the National Research Ethics Service (Ref: 219816) and local departmental research ethics committees. Assessments were face to face, by telephone, by post or online, dependent upon participant preference. In-person contact was required at baseline and at months 9 and 15 in order to complete the interview-based measures. At 9 months the questionnaire on experiences of the study was completed and a subset of participants completed the semi-structured interview.

#### Randomisation, concealment of allocation, and blinding

Participants were randomised on a 1:1 ratio, with minimisation by trial site and medication status (any medication for Bipolar disorder prescribed: yes/no). To ensure concealment, eligible participants were randomised via a validated password website hosted by Exeter Clinical Trials Unit. The first ten participants were allocated using simple randomisation; the remainder were allocated using the minimisation procedure, maintaining a stochastic element to the algorithm to allow concealment. Participants were randomised prior to the start of each therapy group, and informed by an unblinded researcher.

Researchers conducting follow-up assessments were blind to allocation and participants were reminded of this at each follow-up contact. All unblindings were recorded and where possible, researchers who remained blinded to that participant’s status conducted future follow-up assessments. All statistical analyses were performed by a statistician using groups indicated by an anonymised code.

#### Statistical and economic methods

The primary analyses were performed after 9-month follow-up. Further analyses were performed after the final 15-month follow-up. No interim analyses were conducted. All analyses were on an intention-to-treat basis; included outcomes were reported according to randomised allocation, regardless of the treatment actually received. No attempts to address missing data, such as multiple imputation, were made. The economic analysis took the NHS and social care perspective.

At 9-month follow-up only, an inferential analysis was performed for continuous outcomes only, reporting the relevant 95% CI for the between-group mean difference (intervention minus TAU), but no p value. Inferential analyses included the randomisation covariates of site and baseline medication status.

#### Qualitative analysis

Qualitative interviews and written responses on the feedback questionnaires were analysed using a framework approach (Ritchie and Spencer 2002). A preliminary set of a priori themes was drawn up, based on interview/questionnaire questions (e.g. expectations of therapy; most helpful aspects; unhelpful aspects). Data were entered onto a spreadsheet, creating a matrix of rows (participants) and columns (themes) and populating it with summarised responses. During the process, higher-order themes and sub-themes were inductively generated. These were then tabulated, with exemplary quotes and analytic notes, and visually mapped to explore relationships between them.

## Results

### Feasibility outcomes

#### Recruitment and participant flow

Recruitment took place over an 11 month period between August 2017 and July 2018, with follow-up ending in October 2019 after the final piece of data had been collected.

**Fig. 1:**
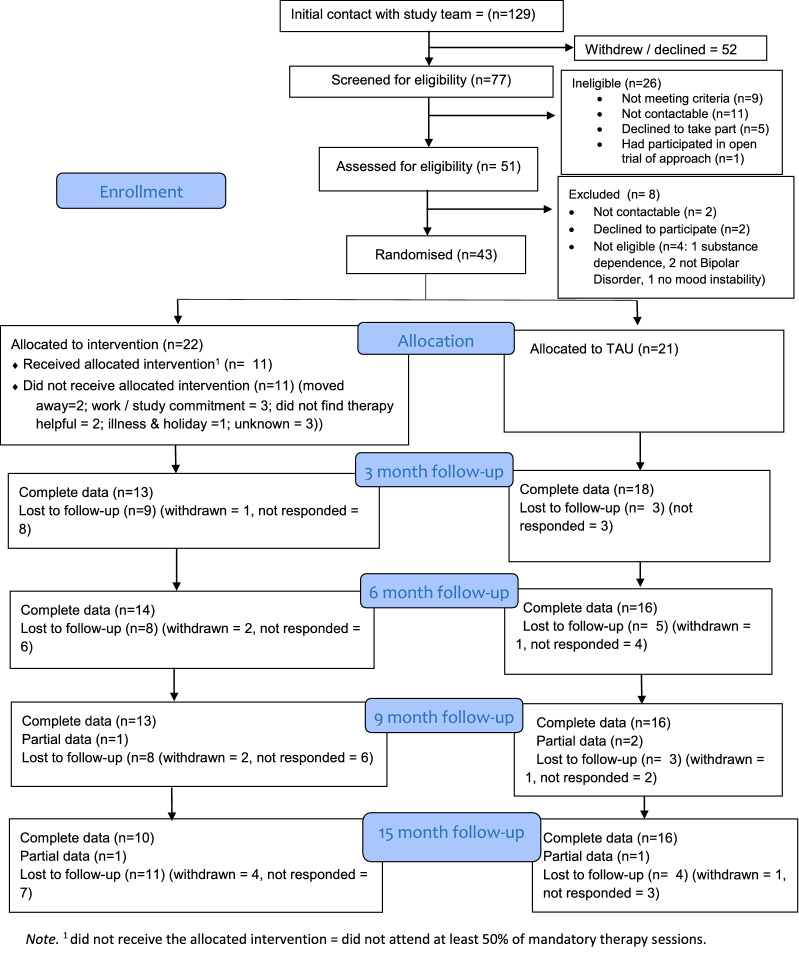
CONSORT diagram of trial recruitment and retention

Recruitment and retention outcomes are shown in Fig. 1. Of 129 individuals who had initial contact with the research team, 77 (60%) were contactable for and completed the screening telephone call. Following this, 9 did not meet criteria, 11 were not contactable afterwards, 5 declined to participate and 1 had already received a previous version of the approach. A total of 51 potential participants were assessed for eligibility. Following this, four were not eligible, two were not contactable and two declined to participate. Of the 43 people randomised 22 were allocated to the intervention arm and 21 to the TAU arm. Recruitment was above target at one site (Devon: 32 planned, 34 actual) and below at the other (Cumbria: 16 planned, 9 actual), resulting in an overall number slightly below our original target (43/48).

The overall randomisation rate for the study was 3.9 participants per month. Within the two sites, this was 3.8 per month in Devon (34 participants over 9 months) and 1.3 per month in Cumbria (nine participants over 7 months).

Of those randomised 33 (77%) were informed about the study by their GP, 5 (12%) through contact with local mental health services, 4 (9%) through media and social media campaigns and 1 (2%) unknown.

Of those randomised to the intervention arm, 8 were allocated to the first Devon group, 9 to the second, and 5 to the group in Cumbria.

### Baseline data

The demographic and clinical characteristics of the sample are given in Table 1. Of 43 participants, 23 (53%) were female, with a median age of 44 years (range 20 to 75 years). The majority of participants met research diagnostic criteria for Bipolar I disorder with the remainder meeting criteria for Bipolar II disorder. In terms of characterising the usual psychiatric care for our sample, of the 40 participants providing detailed information on this for the 6 months prior to study entry, 19 (48%) had contact with a Community Mental Health Team, 1 (3%) reported a psychiatric admission, 10 (25%) reported contact with crisis services and 18 (45%) reported contact with a therapist, counsellor or psychologist. The majority of the sample (38/43: 88%) were prescribed medication for Bipolar disorder at study entry, and most participants were seen in primary care only for their mental health care.

**Table 1:** Demographic and clinical characteristics of trial participants

	ThrIVe-B (n = 22)	Treatment as usual (n = 21)	Total (N = 43)
Gender; n (%)			
Female	14 (64)	9 (43)	23 (53)
Male	8 (36)	12 (57)	20 (47)
Age in years			
Mean (SD)	43.6 (13.0)	49.3 (14.5)	46.4 (13.9)
Median [min, max]	43 [20, 68]	51 [24, 75]	44 [20, 75]
Ethnic group; n (%)			
White	22 (100)	20 (95)	42 (98)
Asian	0 (0)	1 (5)	1 (2)
Prescribed medication for			
Bipolar disorder; n (%)	19 (86)	19 (90)	38 (88)
Mood stabiliser	14 (63)	12 (57)	26 (61)
Antipsychotic	10 (46)	14 (67)	24 (56)
Antidepressant	7 (32)	8 (38)	15 (35)
Site; n (%)			
Devon	17 (77)	17 (81)	34 (79)
Cumbria	5 (23)	4 (19)	9 (21)
In ongoing secondary care; n (%)			
Yes	2 (9)	4 (19)	6 (14)
No	20 (91)	17 (81)	37 (86)
Age of depression onset^a^; mean (SD), n; median [min, max]	19.4 (12.6), 19 16 [6, 58]	18.7 (8.4), 20 18 [6, 44]	19.1 (10.5), 39 18 [6, 58]
Age of mania onset; mean (SD), n; median [min, max]	21.1 (7.9), 15 20 [10, 35]	16.5 (5.7), 17 18 [6, 25]	18.7 (7.1), 32 18 [6, 35]
Bipolar I disorder; n (%)	19 (86)	18 (86)	37 (86)
Bipolar II disorder; n (%)	3 (14)	3 (14)	6 (14)

^a^Minimum age set to 6 years

At study entry participants reported mean depression and anxiety levels in the moderate range, and low levels of current mania symptoms (Table 2). Mean quality of life (QoL.BD) and sense of personal recovery (BRQ) scores for the sample were below but within one SD of those reported in the literature for individuals with Bipolar disorder (Bech et al. 1978; Hamilton 1986).

**Table 2:** Scores on candidate outcome and process measures at baseline and follow-up

Outcome^b^	Baseline		3-month follow-up		6-month follow-up		9-month follow-up			15-month follow-up		SMC (Med, range)
	ThrIVe-B	TAU	ThrIVe-B	TAU	ThrIVe-B	TAU	ThrIVe-B	TAU	ThrIVe-B–TAU^c^ (95% CI)	ThrIVe-B	TAU	
PHQ-9^a^	15.5 (6.6) n = 21	12.9 (5.2) n = 20	10.4 (5.4) n = 12	11.4 (6.1) n = 16	10.6 (5.6) n = 14	12.3 (7.7) n = 17	10.3 (6.5) n = 12	9.8 (4.9) n = 17	− 0.2 (− 4.6; 4.2)	10.7 (7.2) n = 11	8.9 (5.5) n = 17	2 (0–20) n = 20
BRQ^a^	2036 (342) n = 19	1923 (369) n = 16	1969 (503) n = 13	1988 (416) n = 17	1951 (493) n = 11	1927 (427) n = 14	2124 (327) n = 13	1886 (404) n = 16	349 (107; 591)	2238 (483) n = 9	1929 (350) n = 16	447 (2–1903) n = 19
QoL-BD^a^	33.2 (7.5) n = 20	36.6 (6.8) n = 20	34.2 (9.7) n = 13	34.0 (7.9) n = 17	34.1 (8.3) n = 14	34.1 (9.6) n = 17	35.1 (8.3) n = 14	34.0 (10.7) n = 17	2.4 (− 5.2; 9.9)	35.0 (10.7) n = 10	34.5 (9.8) n = 16	4 (0–38) n = 20
ALS total^a^	37.3 (6.1) n = 22	31.5 (13.1) n = 21	23.8 (13.3) n = 13	27.2 (11.7) n = 15	24.9 (8.1) n = 14	27.0 (12.0) n = 17	26.0 (13.5) n = 14	23.5 (12.2) n = 17	− 1.6 (− 8.4; 5.3)	26.6 (15.2) n = 10	21.6 (11.1) n = 16	5 (0–37) n = 21
ALS depression-elation subscale	17.5 (3.3) n = 22	14.9 (6.7) n = 21	10.2 (6.0) n = 13	12.9 (5.7) n = 15	10.8 (5.4) n = 14	13.3 (5.8) n = 17	12.8 (6.8) n = 13	12.2 (5.5) n = 17	− 1.5 (− 4.6; 1.6)	13.1 (7.1) n = 10	11.1 (5.7) n = 16	–
GAD-7	11.2 (4.7) n = 19	10.4 (5.1) n = 20	11.3(4.9) n = 13	9.2 (5) n = 17	11.5 (5.1) n = 14	8.1 (4.6) n = 17	9.3 (5.4) n = 14	7.5 (5.9) n = 17	1.4 (− 2.9; 5.6)	7.8 (4.5) n = 10	8.7 (7.3) n = 16	2 (0–11) n = 20
HDRS	12.8 (8.1) n = 22	6.6 (4.2) n = 19	NR	NR	NR	NR	8.5 (4.8) n = 13	9.5 (5.8) n = 16	− 2.6 (− 7.6; 2.3)	8.2 (7.8) n = 11	6.1 (3.0) n = 17	–
Mean SRM	5.1 (2.9) n = 17	4.6 (2.1) n = 17	5.6 (0.8) n = 11	5.5 (2.8) n = 14	3.9 (2.0) n = 10	3.6 (2.1) n = 12	5.5 (1.9) n = 11	4.9 (1.0) n = 10	0.5 (− 1.5; 2.5)	5.0 (1.2) n = 8	5.5 (0.8) n = 13	–
BMRS	1.8 (2.4) n = 21	1.7 (1.8) n = 21	NR	NR	NR	NR	1.8 (3.2) n = 13	1.9 (2.5) n = 17	0.4 (− 1.6; 2.4)	1.8 (3.6) n = 11	1.0 (1.8) n = 17	–
BARS	2.8 (0.7) n = 18	2.9 (0.5) n = 19	NR	NR	NR	NR	3.8 (0.8) n = 10	3.9 (0.4) n = 15	0.0 (− 0.5; 0.5)	3.9 (0.2) n = 8	3.8 (0.8) n = 14	–
MEPS	3.1 (2.4) n = 21	3.4 (2.2) n = 19	NR	NR	NR	NR	3.7 (2.6) n = 11	2.6 (1.2) n = 16	1.6 (− 0.1; 3.3)	4.1 (2.6) n = 11	2.6 (1.4) n = 17	–
UPPS-P	160.9 (23.8) n = 20	164.3 (25.0) n = 19	158.6 (23.3) n = 13	152.8 (23.0) n = 17	156.8 (22.5) n = 13	158.1 (21.9) n = 17	149.5 (25.7) n = 14	153.5 (21.8) n = 17	− 6.7 (− 16.0; 2.6)	147.5 (28.7) n = 10	154.3 (24.8) n = 16	–
BADS	71.4 (23.4) n = 20	78.5 (26.5) n = 19	81.9 (24.5) n = 13	73.5 (25.1) n = 16	74.5 (33.1) n = 12	72.9 (27.4) n = 16	80.2 (21.5) n = 14	75.1 (33.1) n = 17	7.4 (− 13.3; 28.2)	85.6 (30.3) n = 10	76.6 (30.6) n = 16	–
KIMS total	114.5 (19.1) n = 20	114.8 (21.3) n = 20	117.2 (22.7) n = 12	117.1 (24.7) n = 17	116.2 (24.2) n = 13	116.3 (21.9) n = 16	125.4 (22.9) n = 14	117.9 (25.9) n = 16	13.1 (4.9; 21.3)	128.1 (21.3) n = 10	115.4 (25.7) n = 16	–

TAU = Treatment as usual arm; ThrIVe-B = intervention arm; PHQ-9 = Patient Health Questionnaire; BRQ = Bipolar Recovery Questionnaire; QoL-BD = brief Quality of Life in Bipolar Disorder Scale; GAD-7 = Generalised Anxiety Disorder; HDS = Hamilton Depression Rating Scale; SRM = Social Rhythm Metric; BMRS = Bech Mania Rating Scale; BARS = Brief Adherence Rating Scale; MEPS = Means-Ends Problem-Solving task; UPPS-P = Positive and Negative Urgency, Premeditation, Perseverance, Sensation-Seeking impulsive behaviour scales; BADS = Behavioural Activation in Depression Scale; KIMS = Kentucky Inventory of Mindfulness Scale; SMC = smallest meaningful change according to participant report

^a^Candidate primary outcome measure

^b^Outcomes are reported as mean (SD) unless otherwise stated

^c^Between group mean difference adjusted for site, baseline use of bipolar disorder medications and baseline score

### Trial and therapy retention of participants

We distinguish between research follow-up attrition and therapy attrition, as it was possible for participants to complete therapy and then opt not to complete research follow-up assessments, and vice-versa.

At 9-month follow-up 74% of participants (14 in intervention arm and 18 in TAU arm; 24 [71%] in the Devon site and 8 [89%] in the Cumbria site) provided at least partial follow-up data; 11/43 (26%; 95% CI 14 to 41%) were lost to follow up. Five participants withdrew in the period between randomisation and final follow-up (four in the intervention arm; one in the TAU arm); none were withdrawn by the research team. Figure 1 shows the number of participants returning data at each follow-up point.

Of the 22 participants allocated to receive the intervention, 17 attended at least three sessions (in other words, attended beyond the first individual and first group session).^2^ Of the remaining five, two moved out of the area at the start of the intervention period, one had a competing commitment, one decided the intervention was not suited to them, and one did not attend for reasons unknown. Of the original 22 participants, 11 (50%) completed treatment, defined as attending at least half the 18 “mandatory” sessions. In terms of group attendance per session this ranged from 1 to 8.

Data completion for the smartphone app was low: at baseline, 16 participants provided data for at least 7 days (mean days 10.7, SD 15.5). Between months 3 and 9, 10 participants (6 intervention, 4 TAU) used the app at least once; at 9 months 3 TAU participants used the app at least once (no participants in the intervention group used the app at 9 months).

### Acceptability

Quantitative acceptability data were available from 17 participants (8 in the therapy arm, 9 in the TAU arm) at 9 months. Of these, 16 found the research process at least moderately acceptable, with 14/17 at least moderately satisfied. Of the eight participants in the therapy arm who responded, seven rated therapy as “very” or “extremely” acceptable, and were at least “moderately” satisfied with therapy; one found therapy “slightly” acceptable/satisfactory.

Qualitative acceptability data were synthesised from four sources: acceptability surveys at 9 month follow-up (n = 17), qualitative interviews at 9 month follow-up (n = 14, 8 in therapy arm), surveys of participant experience after the baseline assessment (n = 19) and surveys of those who declined to take part or were not eligible (n = 9).

Making a contribution to society, learning more, and having the chance to access a new therapy emerged as key motives for participation. With regard to the experience of being involved, some participants found the volume of measures challenging; participants also highlighted how important it was that the measures felt relevant to them. Having a positive relationship with the research team was seen as important and was associated with a positive experience of interviews. Participants in the control group expressed some disappointment at not being offered therapy, whilst appreciating of the need for a control group in trials. Barriers to taking part in the study included cost of travel and childcare, and the level of commitment required. The smartphone app was used as part of both the research and therapy aspect of the study. Interruption of activities due to the alerts and direction of attention to negative feelings whilst making mood ratings were seen as downsides and several suggestions were made for improvement. Some participants reported technical issues with the app which may have contributed to low rates of data return.

Analysis of qualitative feedback on the therapy itself (n = 8 interviews, n = 8 surveys) was organised under three main themes: interpersonal aspects of therapy (subdivided into group process and therapist style); therapy content and delivery modes, and where/how the therapy helped. A summary of themes and sub-themes with exemplar quotes is given in Additional file 4.

Participants valued the closed nature of the group (that the same group of people met each session) and the mutual support it generated, although some struggled with particular group dynamics. Both the group and the therapist were instrumental in enabling participants to feel held and understood, which was key to a positive therapeutic experience. Therapist personality and style was seen as very important; authenticity was particularly valued and helped participants to feel that the material was coming from a person rather than from a textbook: “Yeah, [therapist] would give examples from her life which you felt were very genuine … it felt very genuine, and I could get on with that” (201: 4,6). The amount of information delivered was seen to be high; some found this problematic and were worried about overload, but reported that it was helped by the use of multiple modes of delivery and tools for repeating and retaining information, such as handouts and booster sessions. Participants noted the importance of feeling that the material was relevant and tailored to them, and that it was offered at the right time of life. Participants noted several areas of benefit from the therapy, including greater understanding of the self, including re-evaluation of the self in a more positive or accepting way; greater understanding between the self and others; changes in behavioural responses to moods (even if the mood did not change), and broadening their range of activities.

Local clinicians (n = 8) saw DBT-informed therapy as appropriate for the client group, similarly the length of treatment and point of delivery in the care pathway. Clinicians queried whether a group based approach could be delivered and accessed successfully in the health service, as patients might initially be put off by the prospect of a group-based approach, and whether extreme mood states might interfere with group work. Feedback from therapists (n = 4) revealed overall high satisfaction with the techniques and materials, and with the potential to help this patient group, but there was also a sense of having a great deal of information to communicate to patients.

With regard to rankings of personal importance of the candidate primary outcome measures and GAD-7, the mean rankings were highest for the BRQ and ALS and lowest for the PHQ9. The GAD7 was ranked number 1 by the greatest proportion of participants (8/30: 27%), followed by the BRQ and ALS (both 7: 23%). The median smallest meaningful change values for each of the five measures are given in Table 2: for each measure, values given by participants were widely distributed. This may represent genuine lack of consensus but may also suggest that further refinement of the process of obtaining smallest meaningful change values is required in this population.

### Study and therapy safety

Over the study period there were 48 adverse events (8 prior to randomisation, 19 in the therapy arm and 18 in the TAU arm post randomisation). These included 32 instances related to risk of suicide or self-harm (including enactment of study risk protocol following reports of suicidal ideation, and any reported acts of self-harm or towards ending one’s life), six reports of distress during or after study procedures, eight physical health events requiring treatment and two psychiatric admissions. Five were serious adverse events, none of which were unexpected. It was judged that trial procedures (completion of questionnaires) may possibly have contributed to one of these events (low mood culminating in admission to hospital); the other serious adverse events were judged unrelated to the trial.

### Candidate primary clinical outcomes

Mean scores on candidate primary and secondary outcome measures at follow-up are given in Table 2. At 9 months, the between group mean difference for BRQ was 349 (95% CI 107 to 591), with the intervention arm scoring higher (indicating greater personal recovery). The 95% CI included the minimum clinically important difference score of 200 found by the measure developers (Jones, personal communication), and excluded the null treatment effect. For the PHQ-9, there was minimal between group difference (− 0.2 (95% CI − 4.6 to 4.2)); the between-group differences for the ALS (− 1.6 (95% CI − 8.4 to 5.3) and QoL.BD (2.4 (95% CI − 5.2 to 9.9)) favoured the intervention arm but for all three measures the 95% CIs included the null treatment effect.

At 9-month follow-up, the between group mean difference for the Kentucky Inventory of Mindfulness Skills was 13.1 (95% CI 4.9 to 21.3), indicating higher self-reported mindfulness skills in the intervention group, with the 95% CI excluding the null treatment effect. The remaining nine secondary outcomes all yielded a 95% CI that included the null treatment effect.

Completion rates were similar between EQ-5D-3L and SF-36 measures, but lower for the resource use measure. Table 3 displays aggregated data for the health states utility values derived from the EQ-5D-3L and the SF-36. At 9-months, the arms differed significantly with respect to SF-36 utility scores (0.11; 95% CI 0.01–0.20), favouring in the intervention arm. At 9 months no significant difference between arms was observed in health care resource use (Additional files 5 and 6). The cost of providing the ThrIVE-B intervention per participant was £806.29 (Additional file 7).

**Table 3:** Health states utility value scores at baseline and follow-up

Outcome^a^	Baseline		3-month follow-up		6-month follow-up		9-month follow-up			15-month follow-up	
	ThrIVe-B	TAU	ThrIVe-B	TAU	ThrIVe-B	TAU	ThrIVe-B	TAU	ThrIVe-B–TAU^b^ (95% CI)	ThrIVe-B	TAU
EQ-5D-3L	0.52 (0.30) [− 0.18, 1] n = 20		0.65 (0.25) [0.08, 1] n = 13	0.59 (0.36) [− 0.18, 0.85] n = 14	0.51 (0.31) [− 0.02, 1] n = 14	0.65 (0.33) [− 0.18, 1] n = 16	0.63 (0.29) [0.08, 1] n = 14	0.63 (0.35) [− 0.08, 1] n = 17	0.15 (− 0.12, 0.41) n = 28	0.64 (0.31) [0.08, 1] n = 10	0.57 (0.35) [− 0.02, 1] n = 16
SF-36	0.58 (0.15) [0.26, 0.85] n = 20	0.64 (0.11) [0.46, 0.86] n = 20	0.63 (0.12) [0.39, 0.81] n = 13	0.63 (0.12) [0.40, 0.81] n = 17	0.63 (0.10) [0.50, 0.86] n = 14	0.64 (0.13) [0.40, 0.86] n = 17	0.64 (0.14) [0.37, 0.88] n = 13	0.60 (0.13) [0.32, 0.88] n = 16	0.11 (0.01, 0.20) n = 29	0.65 (0.12) [0.43, 0.85] n = 10	0.62 (0.11) [0.45 0.88] n = 15

^a^Outcomes are reported as mean (SD) [range] unless otherwise stated

^b^Adjusted for site, base use of bipolar disorder medication and baseline score

## Discussion

This randomised, controlled feasibility trial is the first to focus specifically upon people with ongoing mood instability within Bipolar Spectrum Disorders, and represents an important step in the development and testing of a novel psychological intervention for this population. In accordance with the study aims, we established recruitment pathways and trial teams in two sites and gathered information on patient recruitment and attrition rates. The overall rate of randomisation of 3.9 participants per month supports the feasibility of a larger trial, recruiting across a longer period and across multiple sites. This could be maximised by learning from experiences in the current trial, where one site recruited more successfully than the other. In particular, rural sites face greater challenges than do more urban ones when aiming to fill a therapy group with patients who have a relatively low-prevalence condition. In this study we initially sought to recruit patients from primary care only; whilst this was the case for the majority of participants, a small number were in secondary care. Setting out to work with a larger number of GP practices per site, selecting sites with a track record of primary care mental health recruitment and including a generally publicised self-referral option should be a priority for future studies in this population.

All four candidate primary outcome measures were seen as similarly valuable by participants and had similar rates of completion: on this basis any of these could be suitable as a primary outcome measure in future trials. Based on participant feedback about key areas of perceived change, it may be appropriate to select two primary outcome measures, one reflecting mood variability and another functioning or recovery. Rates of completion were similar across health economics measures.

Continuation criterion one (no unexpected serious adverse reactions to the therapy or research procedures) was met. Completion rate of measures met continuation criterion three (follow up data at 9 months are available from at least 60% of participants), with no evidence of greater attrition in the control arm. Nevertheless some participants in both arms reported finding the self-report measures onerous; this could potentially be improved by reducing the number of measures, using a more sophisticated online data collection platform, and making more use of participant payment. Whilst participants reported many positive aspects of the therapy, overall therapy attendance rates were numerically lower than the previous open trial (Wright et al. 2020), which may be the result of the imprecision inherent in estimates derived from small samples, but may also request variation in ease of recruitment in this multi-site trial. Nevertheless, as they were also below those specified a-priori in continuation rule four (at least 60% of patients complete at least 50% of sessions) improvement of the therapy content and delivery is required. Using a one-to-one rather than group-based method of delivery would increase accessibility to patients due to flexibility in scheduling and the potential to conduct sessions by telephone when patients are unable to attend in person. Feedback from participants suggested that whilst there were some benefits of group-based delivery, therapist factors played a significant role in successful engagement with the therapy content, thus supporting the feasibility of one-to-one delivery. Responding to feedback from participants about the high level of information provided and the importance of material being personally tailored, future iterations should focus on simplifying the therapy content and working out how to best tailor it to participants’ situations and stage in life.

The study was not designed to test efficacy thus conclusions about the effects of the therapy cannot be drawn from the outcome data, however from the pattern of results there was no strong indication of a detrimental effect of therapy, and where there were significant differences between groups these favoured the therapy arm.

As an assessment of feasibility, a limitation of the current study is that qualitative feedback about the therapy and trial procedures was not gathered from all participants, with fewer than half the original sample completing the acceptability survey at 9 months. In particular, it is possible that those returning written feedback or participating in interviews may be those with a more positive view of the study.

Following modifications to the therapy protocol, further feasibility testing will be necessary to establish whether these result in increased therapy attendance, before progressing to a definitive trial of the approach.

In conclusion it is broadly feasible to conduct a trial of this design within the population of people with frequent bipolar mood swings; our findings suggest several areas of improvement that could be made to enhance recruitment and retention and to increase therapy accessibility and acceptability.

## Supplementary Information

**Additional file 1.** Logic model for ThrIVe-B programme.

**Additional file 2.** Screenshots displaying elements of the ThrIVe-B App.

**Additional file 3.** Table displaying administration schedule for study measures.

**Additional file 4.** Table of themes relating to the intervention, arising from qualitative analysis of interview transcripts (n = 8) and feedback surveys (n = 8).

**Additional file 5.** Table displaying intervention costing.

**Additional file 6.** Table displaying unit cost of services.

**Additional file 7.** Table displaying cost of services by participant (average number of contacts).

## Data Availability

The datasets generated and analysed during the current study are not publicly available due to the sensitive nature of the data but are available from the corresponding author on reasonable request.

## Footnotes

1 The National Institute of Health and Clinical Excellence preferred health-related quality of life measure in adults for economic evaluations is EQ-5D. This study used the 3-level instead of the 5-level version stated in the original protocol.

2 One participant in the TAU arm unintentionally received one session of ThrIVe-B content. They remained in the study.
