# Supplementary material for: Psychological therapy for mood instability within bipolar spectrum disorder: a randomised, controlled feasibility trial of a dialectical behaviour therapy-informed approach (the ThrIVe-B programme)
Source: Int J Bipolar Disord. 2021 Jul 1;9:20. doi: 10.1186/s40345-021-00226-4 (PMC8245616; doi:10.1186/s40345-021-00226-4)
Supplement: Supplementary file 1 — Additional file 1. Logic model for ThrIVe-B programme. [file 40345_2021_226_MOESM1_ESM.docx]

Additional File 1: Logic Model for ThrIVe-B Progamme

| **Thrive Programme Components and Goals** | **Programme modalities: specific strategies, techniques and methods used to accomplish goal** | **Factors maintaining distress and impairment.** | **Expected mediators (and assessment of these in current study)** | **Expected outcomes (and assessment of these in current study)** |
| --- | --- | --- | --- | --- |
| **Goal of Thrive-BD is to give people who experience frequent bipolar mood swings the skills to notice emotion-driven states of mind, and to make a choice of subsequent action that is strategic, in that it fits both the needs of the current context and the person’s own goals and values. Ultimately it is expected that this will result in reduced distress, increased satisfaction with daily functioning, and reduced mood variability.** | | | | |
| Five main components:  **Group skills training**, including the following modules:  ***Mindful awareness*:**  Being deliberately aware of internal and external experience, and meeting this experience without judgement.  Component 1 builds the scaffolding for later components, in that it provides participants with the skills to notice and decentre from internal and external events that typically prompt emotion-driven behaviour. Regular use of mindfulness skills also provides opportunity for graded emotional and experiential exposure. | Mindfulness module (sessions 1 & 2) and refresher session (9). Use of concept of “states of mind”, “what” and “how” skills of mindfulness.   - Experiential practice and coaching. - Discussion in relation to personal examples. - Exploring interaction between use of mindfulness skills and current mood state. - Opening mindfulness practice at each of the 15 sessions; across the programme progresses through exposure to external events, body states, emotions and interpersonal experiences. | Note: the causes of distress, impairment and frequent mood swings within BD are seen as heterogenous and varying across individuals, therefore not all factors below are equally significant for all individuals.   - - Tendency to dysregulation of approach motivation and circadian rhythms in the face of stressors of these systems (targeted by lifestyle and routine / activity stabilisation, also all components should reduce stressors that may lead to dysregulation). - - Reduced inhibitory control and / or difficulties with sustained attention, trait or mood-state linked (targeted by mindful awareness, tolerating intense moods and emotion regulation modules). - - Affective coping behaviours that promote dysregulation and functional / interpersonal difficulties (rumination, avoidance, channelling, stimulation seeking) (targeted by mindful awareness, tolerating intense moods and mood regulation modules, app) - - In response to repeated mood dysregulation and its consequences, emotional avoidance / suppression: leads to dysphoria, and flatness of affect (targeted by emotional exposure via mindfulness and emotion regulation practice, as well as scheduling individual and interpersonal activities, app). - - In response to ongoing mood dysregulation and its consequences, reduced change to learn / practice and have reinforced skills in problem-solving, interpersonal communication, conflict resolution (targeted by problem-solving, wise relationships skills, app) - In response to repeated mood dysregulation and its consequences, shame: increases interpersonal isolation, reduces help seeking.(targeted by group element of programme, mood regulation, wise relationships / reclaiming social territory, individual sessions promote help seeking through facilitating group attendance, supporters’ session). - - Contextual factors reflecting result of ongoing mood dysregulation and its consequences: reduced social support, reduced sources of positive reinforcement (targeted by wise relationships, patterns that promote wise mind, supporters’ session). | Increases in:  - trait mindfulness  - Acceptance of emotion  - social and emotional problem-solving  -routine stability  -sense of “fit” with therapy  Decreases in:  - Avoidance /Rumination  -Social Avoidance  -mood-linked impulsivity | Increases in:  -sense of personal recovery  - quality of life  Decreases in:  -anxiety  - depressive symptoms  - clinically significant hypomanic symptoms  -mood variability |
| ***Day-to-day mood regulation:***  Managing both the contextual factors that contribute to extreme mood states, and one’s behavioural response to these states. | Emotion regulation module (sessions 3-8): promoting wise mind through lifestyle, responses to emotion (going with, letting go, acting opposite),doing what is necessary (problem solving), using emotion regulation in high and low states, activity patterns that promote wise mind (routine and activity scheduling). |  |  |  |
| ***Tolerating intense moods (akin to “distress tolerance” in standard DBT):***  Learning optimal short-term strategies to tolerate extreme mood states. Recognises that for some individuals, complex, strategic action can be blocked by extreme mood states, thus describes simple, functional, emotional coping strategies. | Distress / Activation tolerance techniques: body, soothe, distract, improve, accept). |  |  |  |
| ***Wise relationships*** Bringing mindful awareness and strategic action to interpersonal relationships. | Mindful awareness within interactions (awareness of emotion and action urge, awareness of other’s state of mind, choosing wise action), balancing internal and external awareness (re-directing self-focussed attention). Getting your needs met, repairing / improving relationships, reclaiming social territory. |  |  |  |
| **Individual sessions**  These following a treatment hierarchy:  1. life threatening behaviours  2. therapy interfering behaviours  3. using skills to manage immediate dysregulation / distress  4. tackling less immediate difficulties  5. thriving (keeping positives going, building resilience and meaning) | Stage 1: goal setting, facilitating group attendance, conceptualising participant’s own experience within the explanatory framework of the Thrive model.  Stage 2: coaching participant to use skills learned in group.  Stage 3: Maintenance and resilience building (struggling, coping, thriving framework). |  |  |  |
| **Smartphone Application (App)**  This is present to promote mindful awareness in everyday life, and generalisation of skills from group sessions to everyday life. | Stage 1: self-rating mood when prompted by app. User can select number of alerts per day. Requires individual to interrupt ongoing activity, observe and describe current mood state.  Stage 2: User defines a threshold for low and high mood, above / below which the app displays a message, pre-set by the user. This allows the individual to bring skills learned into moments of heightened emotion. This may lead to i) more effective response to heightened emotion in that instance, and associated positive consequences; ii) over time, learned tendency to reflect before acting during extreme mood states. |  |  |  |
| **Supporters’ Session**  Supporters of the participant (e.g. partner, friend, family member) can attend this session with the aim of learning how to support their friend / relative with concepts and techniques covered in the programme. | Session covers: i) Bipolar Disorder and Bipolar Mood Swings; ii) key concepts and techniques covered on the programme and how to support your friend / relative with these; iii) signposting to further information and support. |  |  |  |
| **Booster Session**  This occurs 3 months post therapy, with the aim of facilitating continuation of skills practice. | Session celebrates participants’ use of skills since end of therapy, facilitates future use of these (contexts that support them, positive consequences of using them), revisits core concepts and explores these in relation to issues raised by participants, coaches participants in using skills where there are difficulties. |  |  |  |
