# Supplementary material for: Psychological therapy for mood instability within bipolar spectrum disorder: a randomised, controlled feasibility trial of a dialectical behaviour therapy-informed approach (the ThrIVe-B programme)
Source: Int J Bipolar Disord. 2021 Jul 1;9:20. doi: 10.1186/s40345-021-00226-4 (PMC8245616; doi:10.1186/s40345-021-00226-4)
Supplement: Supplementary file 2 — Additional file 2. Screenshots displaying elements of the ThrIVe-B App. [file 40345_2021_226_MOESM2_ESM.docx]

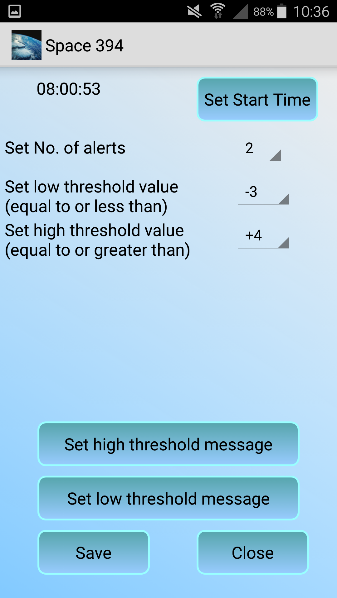

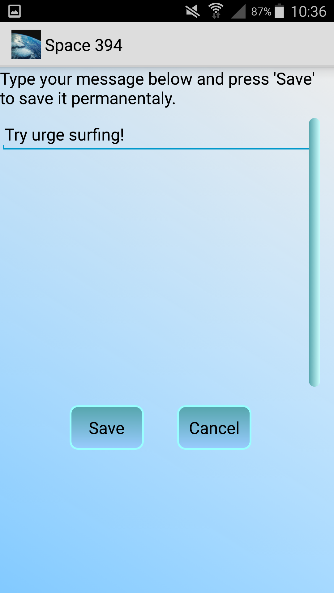
Additional File 2

**
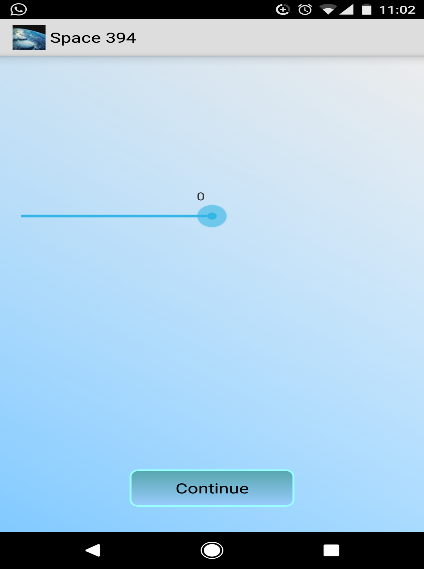
***Screenshots Displaying Elements of the ThrIVe-B App*

*Note*. Screenshot 1 displays the slider scale on which participants are asked to rate their momentary mood (from -10 to +10); 2 displays the screen used for setting mood thresholds [ThrIVe-B group only]; 3 displays the screen used for setting a feedback message if momentary mood is above the high threshold, with example message [ThrIVe-B group only].

3.

2.

1.
