# Supplementary material for: Psychological therapy for mood instability within bipolar spectrum disorder: a randomised, controlled feasibility trial of a dialectical behaviour therapy-informed approach (the ThrIVe-B programme)
Source: Int J Bipolar Disord. 2021 Jul 1;9:20. doi: 10.1186/s40345-021-00226-4 (PMC8245616; doi:10.1186/s40345-021-00226-4)
Supplement: Supplementary file 3 — Additional file 3. Table displaying administration schedule for study measures. [file 40345_2021_226_MOESM3_ESM.docx]

Additional File 3

Table displaying administration schedule for study measures

| **Measure** | **Baseline** | **3 month follow-up** | **6 month follow-up** | **9 month follow-up** | **15 month follow-up** |
| --- | --- | --- | --- | --- | --- |
| Patient Health Questionnaire– 9 | ✓ | ✓ | ✓ | ✓ | ✓ |
| General Anxiety Questionnaire – 7 | ✓ | ✓ | ✓ | ✓ | ✓ |
| Affective Lability Scale | ✓ | ✓ | ✓ | ✓ | ✓ |
| Bipolar Disorder Recovery Questionnaire | ✓ | ✓ | ✓ | ✓ | ✓ |
| Brief Quality of Life in Bipolar Disorder Scale | ✓ | ✓ | ✓ | ✓ | ✓ |
| Health care useage record | ✓ | ✓ | ✓ | ✓ | ✓ |
| **Measure** | **Baseline** | **3 month follow-up** | **6 month follow-up** | **9 month follow-up** | **15 month follow-up** |
| Short-Form-36 item | ✓ | ✓ | ✓ | ✓ | ✓ |
| The EuroQoL 5-Dimension 3 Level | ✓ | ✓ | ✓ | ✓ | ✓ |
| UPPS-P | ✓ | ✓ | ✓ | ✓ | ✓ |
| Behavioural Activation for Depression Scale | ✓ | ✓ | ✓ | ✓ | ✓ |
| Kentucky Inventory of Mindfulness Skills | ✓ | ✓ | ✓ | ✓ | ✓ |
| Adapted Social Rhythm Metric | ✓ | ✓ | ✓ | ✓ | ✓ |
| Brief Adherence Rating Scale | ✓ | X | X | ✓ | ✓ |
| **Measure** | **Baseline** | **3 month follow-up** | **6 month follow-up** | **9 month follow-up** | **15 month follow-up** |
| Bech Mania Rating Scale | ✓ | X | X | ✓ | ✓ |
| Hamilton Depression Scale | ✓ | X | X | ✓ | ✓ |
| Structured Clinical Interview for DSM-V | Sections on mood disorders, psychosis screening section, and substance dependence | X | X | Mood disorders section (since last assessment point) | Mood disorders section (since last assessment point) |
| Means Ends Problem Solving Task | ✓ | X | X | ✓ | ✓ |
| Acceptability Questionnaire | ✓ | X | X | ✓ | X |
| **Measure** | **Baseline** | **3 month follow-up** | **6 month follow-up** | **9 month follow-up** | **15 month follow-up** |
| Qualitative interview (sub-sample of ppts) | X | X | X | ✓ | X |
| One week smartphone app monitoring | ✓ | X | X | ✓ | X |
| Recovery symptom importance ranking & meaningful change | X | X | X | ✓ | X |

*Note.* ✓ = administered at this timepoint; X = not administered at this timepoint; UPPS-P = Positive and Negative Urgency, Premeditation, Perseverance, Sensation-Seeking impulsive behaviour scales.
