# Supplementary material for: Psychological therapy for mood instability within bipolar spectrum disorder: a randomised, controlled feasibility trial of a dialectical behaviour therapy-informed approach (the ThrIVe-B programme)
Source: Int J Bipolar Disord. 2021 Jul 1;9:20. doi: 10.1186/s40345-021-00226-4 (PMC8245616; doi:10.1186/s40345-021-00226-4)
Supplement: Supplementary file 4 — Additional file 4. Table of themes relating to the intervention, arising from qualitative analysis of interview transcripts (n = 8) and feedback surveys (n = 8). [file 40345_2021_226_MOESM4_ESM.docx]

Additional File 4

Table of themes relating to the intervention, arising from qualitative analysis of interview transcripts (n=8) and feedback surveys (n=8)

| **Major theme** | **Subtheme** | **Minor subtheme** | **Example quotation** |
| --- | --- | --- | --- |
| 1. Interpersonal aspects of therapy | 1.1. Group process and content | 1.1.1. Shared experiences | “you feel that you have all gone through the same process together. You’ve built up the same knowledge at the same level, and it can help you to approach the others in the group” (ppt 201) |
|  |  | 1.1.2. Support in making change | “It was a sort of natural environment in which you could explore what was suggested by the therapy” (ppt 202) |
|  |  | 1.1.3. Challenges | “I found the group dynamic very difficult” (ppt 203) |
|  |  | 1.1.4. Group supportive | *sense of camaraderie despite very different characters* (ppt 204) |
|  |  | 1.1.5. Stucture / regularity of meetings | [helpful because of] *weekly support – struggling to manage on my own* (ppt 205) |
|  |  | 1.1.6. Company | [group meeting was helpful because of…] *company* (ppt 206) |
|  |  | 1.1.7. Inspiration | *Seeing certain ideas/ techniques have real positive effect for others* 204 |
|  |  | 1.1.8. Valued closed nature of group | “[appreciate having a closed group because] people entering and leaving on a rolling basis makes for an overwhelming feeling” (ppt 202) |
|  |  | 1.1.8. Disconfirming case | “[rolling group] would make no difference whatsoever to me” (ppt 207) |
|  | 1.2. Therapist stance and style | 1.2.1. Comfortable and safe | “felt umm seen, appreciated, valued, kept safe” (ppt 206) |
|  |  | 1.2.2. Understanding and responsiveness | “They were sympathetic when they needed to be, and sort of not – if you were responding sort of emotionally, they knew when to back off and things” (ppt 208) |
|  |  | 1.2.2 Disconfirming | “I didn’t feel that there was much actual therapeutic connection” (ppt 203) |
|  |  | 1.2.3. Treated me like an equal | “…being spoken to as an equal, rather than being talked down to….. Like being on first-name terms with the therapist and that sort of thing. You felt you were more valued [laughs] as an individual, and your opinion mattered.” (ppt 202) |
|  |  | 1.2.3. Disconfirming | “ [the approach was]… a little systematic, impersonal, occasionally patronising” (ppt 203) |
|  |  | 1.2.4. Made it interesting | “And, that’s what they did here. They made things more interesting you know. And, yeah, I think that’s – that’s one of the keys you know” (ppt 207) |
| 2. Content and delivery | 2.1. Relevance | 2.1.1. Relevant to me | “therapy provided useful information….it was all relevant. It was all, I could relate to a lot of it” (ppt 209) |
|  |  | 2.1.1. Disconfirming: impersonal | “And, it felt as though this was a kind of prescribed process” (ppt 203) |
|  |  | 2.1.2.Techniques relevant to challenges I face | “I’m finding I’m using one of the exercises they call “DEAR”, quite helpful, which is having courageous conversations with people. I’m finding I’m using that quite a bit because umm, I realise that I can let…I’ve only just started to realise how appropriate it is for some of my problems, cos it means that you have a way of diffusing things or managing things in your own control before they get out of control.” (ppt 201) |
|  |  | 2.1.2 disconfirming: not so relevant to extreme states | “….. coping with losing your temper in public situations and things and it all goes wrong. I didn’t feel that it was strong enough to deal with that level of emotion”. (ppt 201). |
|  | 2.2. Getting information across effectively | 2.2.1. Too much information | “[There was] a lot of information to take on-board, and sort of struggled with that” (ppt 202) |
|  |  | 2.2.1 disconfirming | “It was quite in depth…it covered a lot of areas which was quite useful to me in general really” (ppt 201) |
|  |  | 2.2.2. Importance of retaining the information | “It’s good to review it and if you knew that you were going to go back for another top-up, you’ll probably review things again” (ppt 208) |
|  |  | 2.2.3. Pacing (too rushed) | “If [group sessions] were twice as long it would be a lot better” (ppt 202) |
|  |  | 2.2.4. At the right time in life | “If I’d received something like this in my twenties it would have been manna from heaven! It would have just been – it would have been a gift. To me, other people who were the same it would have been a gift. To get some understanding of education strategies would have been amazing. I think now, at my age and with my memory, it’s not too late. It’s never too late. But, I’m not, maybe getting the maximum out of it simply due to my exhaustion of living with this for many years and my memory” (ppt 206) |
|  | 2.3 Strengths and weaknesses of multiple modalities | 2.3.1. Both individual and group sessions necessary | “I think that the therapists and the group experience was fabulous. And, that worked together well for me.” (ppt 206) |
|  |  | 2.3.1. disconfirming | [Individual sessions allowed] *more focus on my situation* (ppt 208) |
|  |  | 2.3.2. Family and friends session not for everyone | “They offered it, but umm my wife worked on the day that err the session was held, and didn’t want to take a day off work”. (ppt 208) |
|  |  | 2.3.3. App needs work | *[The App] didn’t work a lot of the time and therefore questionable how valuable the data can really be (also frustrating that it didn’t work consistently)* (ppt 204) |
| 3. Where and how it was helpful | 3.1. Understanding of the self and others, by self and others | 3.1.1. Self | “I tend to look at myself and kind of – yeah, I analyse myself even more than what I used to do. So, I guess that has to be a good thing”. (ppt 207)  “I’ve been more accepting of the way I am” (ppt 201) |
|  |  | 3.1.2. Others | “Yeah I do try and listen more, that’s a big thing for me. You know, it’s not just me, everybody around me as well that needs to listen, you know if people understand me, then I can understand them. Yeah, so it’s had an effect on not just me, but other people. I try to be more understanding and things like that”. (ppt 209) |
|  | 3.2. Changing the response not the mood |  | “Some of the techniques we’ve been taught, some of them I’ve used quite, in my daily life. They don’t necessarily stop the ups and downs and things like that but they help manage them I think” (ppt 209) |
|  | 3.2. Disconfirming |  | “[the therapy skills]..improved my mood you know, from a depressed state” (ppt 208) |
|  | 3.3. Broadening out |  | “[I am now] ..engaging more in things that help me in other areas beyond therapy” (ppt 208), |
|  | 3.4. Reaffirming existing skills |  | “doing the mindfulness meditation practice at the beginning and ends of the session reminded me how helpful I sometimes find that practice”. (ppt 203) |

*Note*. Ppt = participant identification number for qualitative analysis purposes; quotations in italics represent written feedback from feedback survey questionnaires; quotations in quotation marks represent verbal feedback from qualitative interviews.
