# Supplementary material for: Psychological therapy for mood instability within bipolar spectrum disorder: a randomised, controlled feasibility trial of a dialectical behaviour therapy-informed approach (the ThrIVe-B programme)
Source: Int J Bipolar Disord. 2021 Jul 1;9:20. doi: 10.1186/s40345-021-00226-4 (PMC8245616; doi:10.1186/s40345-021-00226-4)
Supplement: Supplementary file 5 — Additional file 5. Table displaying intervention costing. [file 40345_2021_226_MOESM5_ESM.docx]

Additional File 5

Table displaying intervention costing

| Stage | Resource | Time (hrs) | Unit cost (per hr) | Total (£) | Cost per participant (£) |
| --- | --- | --- | --- | --- | --- |
| Training | Therapist | 40 | £19.74 | 789.6 |  |
|  | Lead therapist | 40 | £25.17 | 1006.8 |  |
|  | Supervisor | 40 | £35.28 | 1411.2 |  |
| *Subtotal* | | | | ***£3,207.60*** | ***£145.80*** |
| Supervision | Therapist | 60 | £19.74 | 1184.4 |  |
|  | Lead therapist | 60 | £25.17 | 1510.2 |  |
|  | Supervisor | 60 | £35.28 | 2116.8 |  |
| *Subtotal* | | | | ***£4,811.40*** | ***£218.70*** |
| Group session^†^ | Therapist | 44 | £19.74 | 868.56 |  |
|  | Lead therapist | 44 | £25.17 | 1107.48 |  |
| *Subtotal* | | | | ***£1,976.04*** | ***£260.32**** |
| *Per group session* | | | | ***£123.50*** |  |
| Individual session^††^ | Therapist | 82 | £19.74 | 1618.68 |  |
|  | Lead therapist | 82 | £25.17 | 2063.94 |  |
| ***Subtotal*** | | | | ***£3,682.62*** | ***£167.39***** |
| ***Per individual session*** | | | | ***£44.91*** |  |
|  | Resource | Number pages | Cost per page | **Total (£)** |  |
| Handout | Participant | 2904 | £0.10 | 290.4 |  |
|  | Therapist | 195 | £0.10 | 19.5 |  |
| ***Subtotal*** | | | | ***£309.90*** | ***£14.09*** |
| **Total cost** | | | | **£13,987.56** | **£806.29** |

^†^2 hrs and 45 mins per group session; ^††^1 hr per session;*Sub-total divided mean session attended by participant; **Individual session cost x mean of individual

sessions attended.
