# Supplementary material for: Psychological therapy for mood instability within bipolar spectrum disorder: a randomised, controlled feasibility trial of a dialectical behaviour therapy-informed approach (the ThrIVe-B programme)
Source: Int J Bipolar Disord. 2021 Jul 1;9:20. doi: 10.1186/s40345-021-00226-4 (PMC8245616; doi:10.1186/s40345-021-00226-4)
Supplement: Supplementary file 6 — Additional file 6. Table displaying unit cost of services. [file 40345_2021_226_MOESM6_ESM.docx]

Additional File 6

Table displaying unit cost of services

| Service | Unit cost | Unit | Source | Page | Comment |
| --- | --- | --- | --- | --- | --- |
| *Primary care and community based services* | | | | | |
| GP at surgery/ health centre | 31 | 9.22 mins | PSSRU 2018 | 127 | Per surgery consultation lasting 9.22 minutes without qualification costs including direct care = £31 |
| GP via telephone | 8.1 | call | PSSRU 2018 | 127 | Average cost per GP consultation via telephone |
| GP at home | 38.76 | Consultation | PSSRU 2015 & 2018 | 176 and 127 | 11.4 minutes per home visit (PSSRU 2015), £3.40 per minute patient contact (PSSRU 2018) |
| Practice nurse at surgery/ health centre | 9.3 | 15.5 mins | PSSRU 2017 & 2018 | 125 | Nurse (GP practice) £36 without qualifications per hour from PSSRU 2018; 15.5 minutes per consultation obtained from PSSRU 2017 as no current information was available in PSSRU 2018 |
| Practice nurse via telephone | 3.96 | call | PSSRU 2017 and 2018 | 125 | Nurse (GP practice) £36 without qualifications per hour from PSSRU 2018; 6.6 minutes per consultation via telephone data from PSSRU 2017 as no current information was available in PSSRU 2018 |
| Physiotherapist at surgery/ health centre | 54 | 1 session | PSSRU 2018 | 89 | cost of 1-to-1, band 6 |
| Occupational therapist at home | 78 | 1 hour | PSSRU 2018 | 89 | 1-to-1 |
| NHS community mental health team | 192 | Per contact | NHS ref costs 17/18 | 7 | Mental health specialist teams, excluding adult IAPT |
| Crisis resolution team | 201.45 | Per contact | PSSRU 2016 | 169 | Adjusted for inflation from £191 in 2015/16 |
| Early intervention team | 161.86 | Per contact | NHS ref costs 10/11 | Spreadsheet | Adjusted for inflation from £143 in 2010/11 |
| Psychological therapy appointment | 53 | 1 hour | PSSRU 2018 | Spreadsheet | Band 7 |
| Psychologist appointment | 53 | 1 hour | PSSRU 2018 | Spreadsheet | Band 7 clinical psychologist |
| Group therapy session | 96 | 1 hour | NHS ref costs 17/18 | Spreadsheet | Other therapist, adult, group |
| Counsellor/ Psychological therapist | 90 | 1 hour | PSSRU 2018 | Spreadsheet | Counsellor consultant, band 8c |
| Nutritionist | 86 | 1 hour | PSSRU 2018 | 89 | Group session with dietician |
| Walk in centre | 44.34 | Attendance | NHS ref costs 11/12 | Spreadsheet | Adjusted for inflation from £40 in 2011/12 |
| A&E Health liaison services | 196 | Per contact | PSSRU 2018 | 35 |  |
| Psychiatrist | 186 | Consultation | NHS ref costs 17/18 | Spreadsheet | Mental health specialist teams, other psychiatric liaison services, adult and elderly |
| Drug and alcohol services | 123 | Contact | NHS ref costs 17/18 | 7 | Non-cluster mental health services |
| *Secondary care services* | | | | | |
| Foot operation | 186.43 | Surgery | NHS ref costs 17/18 | Spreadsheet | Intermediate foot operation, general surgery |
| Psychiatric ward | 270.48 | Day | NHS ref costs 09/10 | Spreadsheet | Acute NHS psychiatric ward. Adjusted for inflation from £270.48 in 2009/10 |
| Outpatient | 125 | Attendance | NHS ref costs 17/18 | 5 |  |
| Day Case | 742 | Attendance | NHS ref costs 17/18 | 5 |  |
| A&E | 160 | Attendance | NHS ref costs 17/18 | 5 |  |
