# Supplementary material for: Psychological therapy for mood instability within bipolar spectrum disorder: a randomised, controlled feasibility trial of a dialectical behaviour therapy-informed approach (the ThrIVe-B programme)
Source: Int J Bipolar Disord. 2021 Jul 1;9:20. doi: 10.1186/s40345-021-00226-4 (PMC8245616; doi:10.1186/s40345-021-00226-4)
Supplement: Supplementary file 7 — Additional file 7. Table displaying cost of services by participant (average number of contacts). [file 40345_2021_226_MOESM7_ESM.docx]

Additional File 7

Table displaying cost of services by participant (average number of contacts)

|  | ThrIVE-B  mean (SD) [range]  N = 22 | | | | | Treatment as usual  mean (SD) [range]  N = 21 | | | | |
| --- | --- | --- | --- | --- | --- | --- | --- | --- | --- | --- |
|  | **Baseline** | **3-m** | **6-m** | **9-m** | **15-m** | **Baseline** | **3-m** | **6-m** | **9-m** | **15-m** |
| *Primary care and community-based services* | | | | | | | | | | |
| Total mean primary care cost to NHS | £125.06 | £199.61 | £10.46 | £25.95 | £219.29 | £73.71 | £145.38 | £96.32 | £206.74 | £328.17 |
| Difference in mean from baseline | - | £74.55 | -£114.60 | -£99.11 | £94.23 | **-** | £71.67 | £22.61 | £133.03 | £254.47 |
| Difference in mean  (ThrIVE - TAU) | - | - | - | **-** | **-** | £51.35 | £54.23 | -£85.86 | -£180.79 | -£108.88 |
| *Secondary care services* | | | | | | | | | | |
| Total mean secondary care cost to NHS | £93.73 | £5.68 | £0.00 | £5.68 | £168.70 | £59.14 | £102.10 | £220.69 | £76.05 | £337.62 |
| Difference in mean from baseline | - | -£88.05 | -£93.73 | -£88.05 | £74.97 | **-** | £42.95 | £161.54 | £16.90 | £278.48 |
| Difference in mean  (ThrIVE - TAU) | - | - | - | **-** | **-** | £34.58 | -£96.41 | -£220.69 | -£70.37 | -£168.93 |

*Reported under other in questionnaire, setting not stated- assumed GP surgery/ Health centre

†Adjusted for site, base use of bipolar disorder medication and baseline score.
